# Biomarkers in glioblastoma and degenerative CNS diseases: defining new advances in clinical usefulness and therapeutic molecular target

**DOI:** 10.3389/fmolb.2025.1506961

**Published:** 2025-03-18

**Authors:** Fan Bu, Jifa Zhong, Ruiqian Guan

**Affiliations:** ^1^ The Third Affiliated Hospital of Xinxiang Medical University, Xinxiang, Henan, China; ^2^ Heilongjiang University of Chinese Medicine Affiliated Second Hospital, Harbin, China

**Keywords:** neurodegenerative, glioblastoma, biomarker, algorithm, therapeutic molecular target

## Abstract

**Background:**

Discovering biomarkers is central to the research and treatment of degenerative central nervous system (CNS) diseases, playing a crucial role in early diagnosis, disease monitoring, and the development of new treatments, particularly for challenging conditions like degenerative CNS diseases and glioblastoma (GBM).

**Methods:**

This study analyzed gene expression data from a public database, employing differential expression analyses and Gene Co-expression Network Analysis (WGCNA) to identify gene modules associated with degenerative CNS diseases and GBM. Machine learning methods, including Random Forest, Least Absolute Shrinkage and Selection Operator (LASSO), and Support Vector Machine - Recursive Feature Elimination (SVM-RFE), were used for case-control differentiation, complemented by functional enrichment analysis and external validation of key genes.

**Results:**

Ninety-five commonly altered genes related to degenerative CNS diseases and GBM were identified, with *RELN* and *GSTO2* emerging as significant through machine learning screening. Receiver operating characteristic (ROC) analysis confirmed their diagnostic value, which was further validated externally, indicating their elevated expression in controls.

**Conclusion:**

The study’s integration of WGCNA and machine learning uncovered *RELN* and *GSTO2* as potential biomarkers for degenerative CNS diseases and GBM, suggesting their utility in diagnostics and as therapeutic targets. This contributes new perspectives on the pathogenesis and treatment of these complex conditions.

## 1 Introduction

Degenerative central nervous system (CNS) diseases (Xu et al., 2022), such as Alzheimer's disease (AD), Parkinson’s disease (PD), multiple sclerosis (MS), and amyotrophic lateral sclerosis (ALS), as well as neurological tumors, particularly glioblastoma (GBM) (Broekman et al., 2018), represent the most challenging diseases in the field of neuroscience. These diseases not only impose a heavy burden on patients and their families, but also pose significant pressures on society and the healthcare system (Wareham et al., 2022).

Despite the significant differences in clinical presentation and pathology between degenerative CNS diseases and GBM, they exhibit remarkable similarities in certain key biological processes, such as ferroptosis, oxidative stress, and neuroinflammation.

Ferroptosis is a form of cell death dependent on iron and lipid peroxidation, shown to play a critical role in both degenerative CNS diseases and GBM. For example, iron accumulation and the resulting oxidative damage are key pathological processes in Parkinson’s disease (Yan et al., 2024). Similarly, iron metabolism abnormalities have been observed in GBM, suggesting that ferroptosis could be a common pathological mechanism in these two types of diseases (Soo et al., 2024). Oxidative stress is important in both degenerative CNS diseases and GBM. Excess reactive oxygen species (ROS) can cause cellular damage and death. In AD and PD, oxidative stress is considered a major cause of neuronal damage (Isidro, 2018). In GBM, oxidative stress not only promotes tumor cell proliferation and migration but may also influence tumor progression by modulating interactions within the tumor microenvironment (Lauryn E et al., 2017). Neuroinflammation is common in degenerative CNS diseases. Inflammation plays a key role in the progression of AD and PD (Pei-Lun et al., 2024). In GBM, the inflammatory environment not only promotes tumor cell growth and survival but may also affect the tumor’s response to treatment by modulating immune cell function (Ana Helena Larangeira et al., 2024).

Research indicates significant overlap in gene and epigenetic regulation between degenerative CNS diseases and GBM. For example, a study (Lu et al., 2023) analyzed the expression of different isoforms (alternative splicing variants) of genes in brain tissue and identified multiple gene regulatory sites associated with neurological traits and diseases. These regulatory sites may play similar roles in different degenerative CNS diseases and GBM. Notably, isoform-ratio quantitative trait loci have been shown to regulate gene expression in brain tissue, with implications for various CNS conditions such as AD, mood fluctuations, and sleep duration. Understanding these regulatory mechanisms across degenerative CNS diseases and GBM could provide new insights into shared molecular pathways and identify novel therapeutic targets (Ashraf et al., 2023). Understanding these common regulatory mechanisms helps elucidate the shared genetic regulation in different diseases. In both brain tumors and degenerative CNS diseases, cell-cell interactions are crucial in disease development and progression. For instance, the activation of astrocytes and microglia is critical in AD progression, where microglial activation contributes to amyloid-β plaque clearance or neuroinflammation, as reported in recent study (Teresa et al., 2024). Similarly, abnormal activation of astrocytes is a key driver of GBM proliferation and invasion, as demonstrated in preclinical research (Jian and Shiwei, 2024). Despite these shared mechanisms, degenerative CNS diseases and GBM exhibit significant differences. Degenerative CNS diseases, such as AD and Parkinson’s disease, are chronic progressive conditions characterized by gradual neuronal damage and death, leading to cognitive and motor function decline (Andrea J. et al., 2020; M. et al., 2024).

In contrast, GBM is a highly invasive malignant brain tumor characterized by rapid growth and extensive damage to surrounding brain tissue. Therapeutically, the treatment of degenerative CNS diseases mainly focuses on symptom management and slowing disease progression, such as using dopamine replacement therapy in Parkinson’s disease. In contrast, GBM treatment includes surgical resection, radiotherapy, and chemotherapy, although these methods often cannot completely eradicate the tumor and it tends to recur.

Despite significant progress in understanding the molecular mechanisms underlying these diseases over the last decades, these findings are far from being fully translated into effective treatments. Therefore, the search for novel biomarkers that can be used as early diagnostic tools, indicators of disease progression, and monitoring means of treatment response, as well as the identification of new therapeutic targets, has become a cutting-edge topic in neuroscience research.

In recent years, the rapid advancements in bioinformatics (Gauthier et al., 2019), genomics (Gangfuß et al., 2022), and proteomics (Cui et al., 2022) have significantly deepened our understanding of the molecular underpinnings of neurological disorders. The application of high-throughput gene expression analyses (Hrdlickova et al., 2017) and differential expression analysis techniques (McDermaid et al., 2019) has emerged as a powerful tool for identifying disease-associated genes and pathways. By examining the gene expression patterns in degenerative CNS diseases and GBM, we aim to uncover potential common molecular mechanisms and cross-disease biomarkers between these conditions. These cross-disease biomarkers not only offer a new perspective for understanding these seemingly disparate diseases, which may share certain fundamental biological traits, but also hold promise for the development of therapeutic approaches that transcend specific diseases.

## 2 Data and methods

### 2.1 Design and methods

**FIGURE 1:**
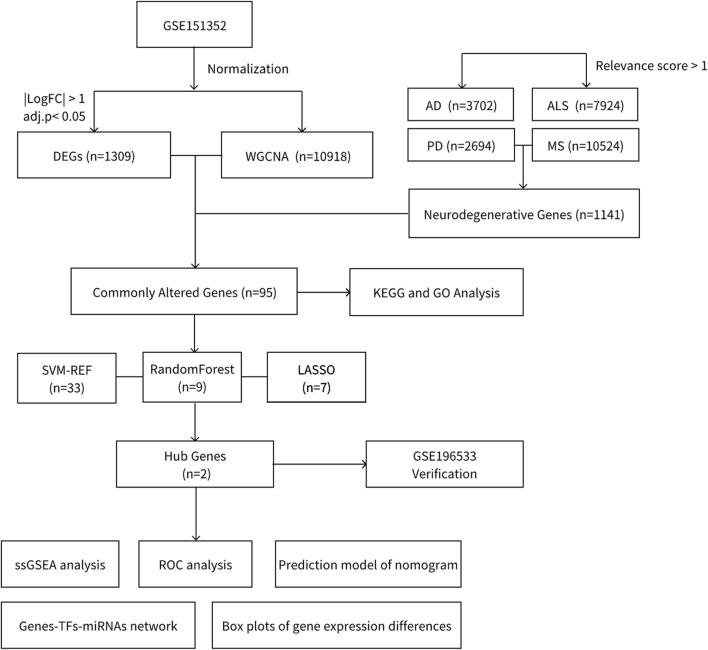
Flowchart.

In this study, we aimed to identify potential biomarkers and pathways implicated in the progression of degenerative CNS diseases and glioblastoma. To achieve this, we integrated intersecting genes from four primary degenerative CNS diseases, sourced from GeneCards, with glioblastoma-related matrices obtained from the Gene Expression Omnibus (GEO). Weighted Gene Co-expression Network Analysis (WGCNA) was performed to identify differentially expressed genes and key modules within these diseases. Subsequently, we used intersecting genes and three machine learning approaches to identify diagnostic genes, *GSTO2* and *RELN*, shared between the two diseases. These genes demonstrated robust diagnostic performance and were validated using external datasets. In addition, we performed Single Sample Gene Set Enrichment Analysis (GSEA) for these genes to identify common pathways associated with degenerative CNS diseases and glioblastoma. Figure 1 provides an overview of the workflow for data preparation, processing, analysis, and validation.

### 2.2 Data acquisition

Genes related to AD, PD, MS, and ALS were retrieved from GeneCards, with a selection threshold set for a relevance score ≥1. The genes at the intersection of these diseases were regarded as representative of the genetic framework for degenerative CNS diseases in our research.

Gene expression matrices for GBM were sourced from the GEO database (GSE151352, Public on 29 May 2020). This dataset was selected due to its focus on paired normal and tumor tissue samples, providing a robust framework for comparing differential gene expression in GBM. Utilizing the platform GPL23934 (Ion Torrent S5), GSE151352 comprises RNA-seq data from 12 GBM patients, each with matched fresh tumor and normal brain tissue samples. The dataset’s design includes rigorous sample validation *via* immunostaining, ensuring the accuracy of tissue classification. These samples were processed using the Ion Ampliseq Transcriptome Human Gene Expression Kit, and sequencing was performed with the Ion S5 semiconductor sequencer. This high-quality dataset provides critical insights into GBM’s molecular heterogeneity, supporting the objectives of this study.

### 2.3 Identification of DEGs

Utilizing R software (version 4.3.1), we identified DEGs of GBM in serum samples from the patient and control groups. Considering the small sample size of our study, we employed the limma package to perform differential expression analysis. Specifically, we first fitted a linear model using the lmFit () function and then applied the empirical Bayes moderation *via* the eBayes () function to adjust for random variance across genes. This moderated T-test approach improves the reliability of DEG identification. For each gene, we compared its average expression between the patient and control groups using a statistical test. This test produced an adjusted p-value, which indicates the likelihood that the observed difference in expression occurred by chance, and a log2 fold change, which quantifies the magnitude of the difference. Genes with an adjusted p-value of 0.05 or less and an absolute log2 fold change of at least 1 were classified as differentially expressed. The log2 scale was applied to make the expression differences easier to interpret and to align with standard practices in gene expression analysis.

### 2.4 WGCNA analysis

To pinpoint pivotal genes, we employed the WGCNA within the R programming environment, focusing on identifying hub genes in highly correlated gene clusters, also known as modules. Initially, we constructed a Topological Overlap Matrix (TOM) to examine the correlations among genes. Subsequently, we calculated the dissimilarity of the TOM (diss TOM = 1 - TOM), which was used to generate a phylogenetic clustering tree. This allowed us to group genes with similar expression patterns into modules through a “TOM-based” approach. We set specific parameters for module aggregation, requiring modules to have at least 10 genes but no more than 500, and applied a clustering height cut-off of 0.25 to ensure clear distinction among modules. Following this, we determined module membership and assessed gene significance, selecting key genes from the primary modules for further analysis.

### 2.5 Commonly altered genes

Utilizing the R programming language, we conducted a filtration process on previously identified genes. This step was aimed at discerning genes at the intersection of three critical categories: genes within GBM’s pivotal WGCNA modules, DEGs in GBM, and commonly altered genes associated with degenerative CNS diseases. This methodological approach enabled us to pinpoint target genes shared across these significant domains, offering potential insights into shared molecular mechanisms and identifying targets for further investigation and therapeutic development.

### 2.6 Enrichment analysis

We identified genes from relevant modules and intersected them with previously determined commonly altered genes to pinpoint commonly altered genes. Subsequently, we utilized R software to perform enrichment analyses on these commonly altered genes, employing both the Kyoto Encyclopedia of Genes and Genomes (KEGG) and Gene Ontology (GO) frameworks.

### 2.7 Hub genes

To single out key candidate genes, we employed three advanced machine learning techniques: Random Forest, Least Absolute Shrinkage and Selection Operator (LASSO), and Support Vector Machine - Recursive Feature Elimination (SVM-RFE). These methods were used to refine the selection of hub genes from those identified as co-expressed, focusing on genes that were consistently highlighted across all three techniques. Each method was applied to prioritize genes based on their relevance and potential as key candidates.

### 2.8 Hub genes validation

To confirm the significance of the identified hub genes, we assessed their diagnostic capabilities through the construction of receiver operating characteristic (ROC) curves and examination of their expression levels within the dataset. To accomplish this, we employed ROC-specific packages in R, facilitating the creation of these curves. We then determined the area under the curve (AUC) for each gene. An AUC value nearing 1 signifies enhanced predictive accuracy, indicating that the gene in question possesses a high potential for distinguishing between disease states. This validation process is crucial for establishing the reliability of our findings, as it directly tests the ability of the hub genes to serve as effective markers for diagnosis. By leveraging the analytical power of R and its ROC-related functionalities, we are able to quantitatively evaluate the diagnostic performance of each gene, ensuring that only those with significant discriminative power are considered in the context of disease identification and analysis.

To validate the diagnostic accuracy of the two identified genes further, we turned to the external dataset GSE196553, which contains data for GBM. This dataset, comprising samples from 9 healthy controls and 61 GBM patients, served as our external validation cohort. We depicted the expression patterns of the diagnostic genes in cohort using boxplots and also computed AUC to assess their diagnostic performance. This step is essential for verifying the reliability and applicability of our findings in a broader context, ensuring that the diagnostic genes maintain their predictive power across different sample sets. By analyzing their expression in an independent group of subjects, we solidify the evidence for these genes’ roles as biomarkers, enhancing confidence in their potential clinical utility.

### 2.9 Regulatory network construction

In our exploration of the hub genes’ functions, we utilized the NetworkAnalyst platform (https://www.networkanalyst.ca/), a dedicated tool for in-depth data analysis, available online. This platform provided us access to essential resources such as the ChEA database, which we used to predict transcription factors (TFs) linked to our hub genes, and the TarBase database, which aided in identifying miRNAs associated with these genes. This thorough methodology allowed us to delineate the intricate network of interactions and regulatory pathways involving the hub genes, offering insights into their roles within biological systems.

### 2.10 ssGSEA of hub genes

The Single-sample Gene Set Enrichment Analysis (ssGSEA) approach is designed to evaluate the presence and activity level of specific gene sets within individual samples, with a particular focus on transcriptomic data. It sorts genes based on their expression levels and generates enrichment scores for selected gene sets. This method is instrumental in identifying potential biomarkers and elucidating biological pathways relevant to disease and therapeutic investigations. In our research, we applied ssGSEA to forecast the expression profiles of key hub genes.

In order to comprehensively analyze gene expression and their correlations with immune data, we first conducted Gene Set Variation Analysis (GSVA) to quantify gene set enrichment scores across different samples. This enabled us to assess the biological variations in gene expression related to specific pathways or processes. Subsequently, we focused on differential expression analysis, comparing the gene expression patterns between the control and disease groups. For correlation analysis, we calculated Spearman correlation coefficients between individual gene expression levels and immune data to identify potential associations. These correlation coefficients and their significance were visualized using heatmaps

This analysis is crucial for understanding how these hub genes function within various biological contexts, enabling us to uncover their roles in disease mechanisms and potential therapeutic interventions. By leveraging ssGSEA, we gain detailed insights into the gene expression patterns that characterize different states or responses, enhancing our ability to pinpoint genes that are critical for disease progression or response to treatment.

## 3 Results

### 3.1 Screening of DEGs

**FIGURE 2:**
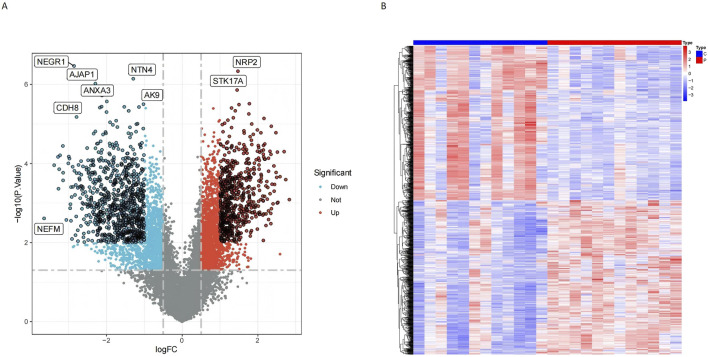
Differential gene expression analysis revealed significant variations between conjunctiva and pterygium. **(A)** Volcano diagram shows that red points indicate upregulated genes, while blue points indicate downregulated genes. Points with black circles represent genes meeting stricter criteria (P < 0.01 and |logFC| > 1), while points without black circles represent genes meeting standard criteria (P < 0.05 and |logFC| > 0.5). **(B)** DEGs heatmap shows the expression of the all upregulated and downregulated genes.

Following the specified screening criteria, a total of 1,309 differentially expressed genes (DEGs) were identified in normal and tumor samples. Among these, 726 genes were downregulated, and 583 were upregulated (Supplementary Table S1). The distribution and characteristics of these DEGs are illustrated in a volcano plot (Figure 2A) and a heatmap (Figure 2B).

### 3.2 WGCNA analysis

**FIGURE 3:**
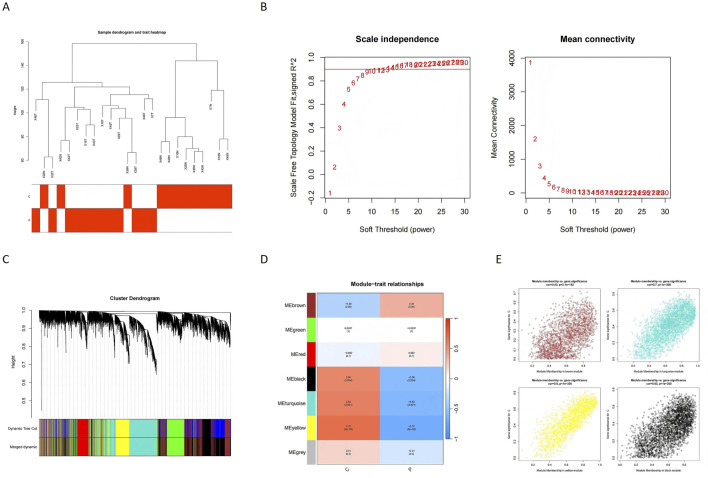
Identification of gene co-expression modules and their relationships with GBM traits. **(A)** The sample dendrogram and trait heatmap show the clustering of samples based on gene expression profiles. The trait heatmap highlights the correlation between samples and glioblastoma traits, aiding in identifying relevant modules for further analysis. **(B)** Scale independence analysis determines the soft threshold power (β) for constructing a scale-free network. The plot shows that a scale-free topology is achieved when β equals 10, ensuring the robustness of the network. **(C)** The dynamic tree cut method is used to identify initial gene modules, which are then merged based on similarity. The plot illustrates the merging of modules to create distinct clusters of commonly altered genes. **(D)** The module-trait relationship heatmap displays the correlation between identified modules and glioblastoma traits. Modules with strong positive or negative correlations are highlighted for further investigation. **(E)** The plot shows the genes assigned to each module and their respective module memberships. This provides an overview of gene clustering and their potential functional relevance.

WGCNA was employed to identify key modules associated with glioblastoma. A sample clustering tree was constructed to visualize the dataset structure (Figure 3A), and the soft threshold was set to 10 to achieve scale-free topology (Figure 3B). After merging similar modules (Figure 3C), seven modules were identified, with their associations to disease traits depicted in Figure 3D. Among these, the MEbrown module showed strong positive correlations, while MEblack, MEturquoise, and MEyellow exhibited significant negative correlations with glioblastoma traits (P < 0.05). These four critical modules collectively encompassed 10,918 genes (Figure 3E; Supplementary Table S2). These modules provided an initial understanding of genes potentially associated with glioblastoma and were used for further analysis.

### 3.3 Screening of genes of degenerative CNS diseases

**FIGURE 4:**
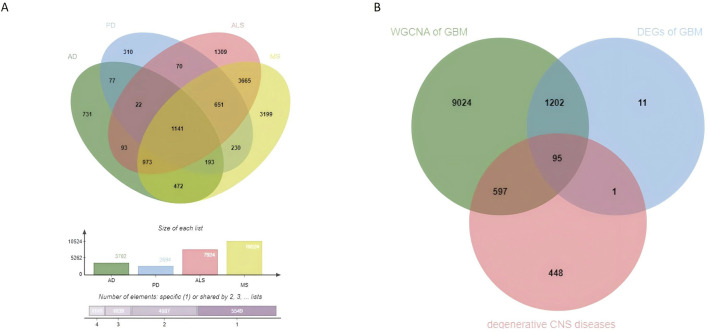
Identification of shared genes between degenerative CNS diseases and glioblastoma. **(A)** Venn diagram shows the overlap of genes associated with AD, PD, MS, and ALS, revealing 1,141 commonly altered genes shared among these degenerative CNS diseases. **(B)** Venn diagram illustrates the intersection of glioblastoma-related WGCNA modules, DEGs, and the shared genes from degenerative CNS diseases, identifying 95 commonly altered genes as potential molecular links between glioblastoma and degenerative CNS diseases.

Using the GeneCards database, genes associated with AD, PD, ALS, and MS were retrieved. Genes with a correlation score ≥1 were selected, and the intersection of these genes yielded 1,141 commonly altered genes for degenerative CNS diseases (Supplementary Table S3; Figure 4A).

### 3.4 Commonly altered genes between GBM and degenerative CNS diseases

To investigate shared molecular mechanisms between glioblastoma and degenerative CNS diseases, we intersected three gene sets: (1) genes from the critical WGCNA modules, (2) DEGs in glioblastoma, and (3) degenerative CNS disease genes. This analysis identified 95 commonly altered genes (Figure 4B). These genes serve as a link between glioblastoma and degenerative CNS diseases, forming the basis for downstream functional and pathway analyses.

### 3.5 Enrichment analysis

**FIGURE 5:**
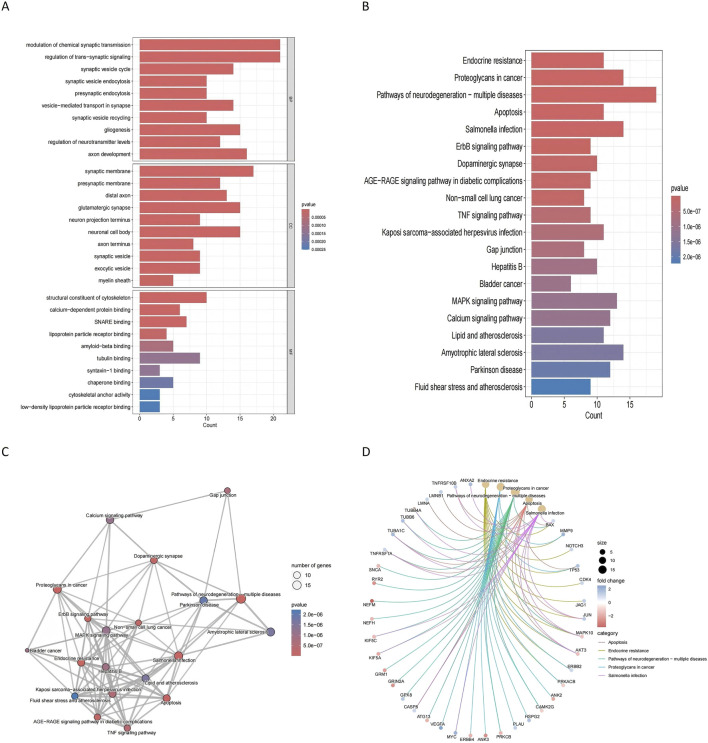
Functional enrichment analysis of commonly altered genes **(A)** GO enrichment analysis shows the biological processes, cellular components, and molecular functions significantly associated with the 95 commonly altered genes. Key enriched processes include synaptic transmission and axon development. **(B)** KEGG pathway analysis identifies significantly enriched pathways. **(C)** KEGG cnetplot visualizes the connections between key pathways and the commonly altered genes. **(D)** KEGG network plot depicts the relationships among enriched pathways, providing insights into the molecular mechanisms linking glioblastoma and degenerative CNS diseases.

Functional enrichment analyses were performed to explore the roles of these 95 genes in glioblastoma and degenerative CNS diseases. Gene Ontology (GO) analysis revealed enrichment in biological processes (e.g., synaptic transmission, axon development), cellular components (e.g., myelin sheath, synaptic vesicles), and molecular functions (e.g., low-density lipoprotein receptor binding) (Figure 5A). KEGG analysis identified pathways such as neurodegeneration, ALS, PD, and proteoglycans in cancer as highly relevant to both glioblastoma and degenerative CNS diseases (Figure 5B). These pathways were visualized through a network diagram to elucidate their interconnected roles (Figures 5C, D). The results of these analyses provide insights into shared molecular mechanisms and highlight potential pathways for further exploration.

### 3.6 Identification of hub genes

**FIGURE 6:**
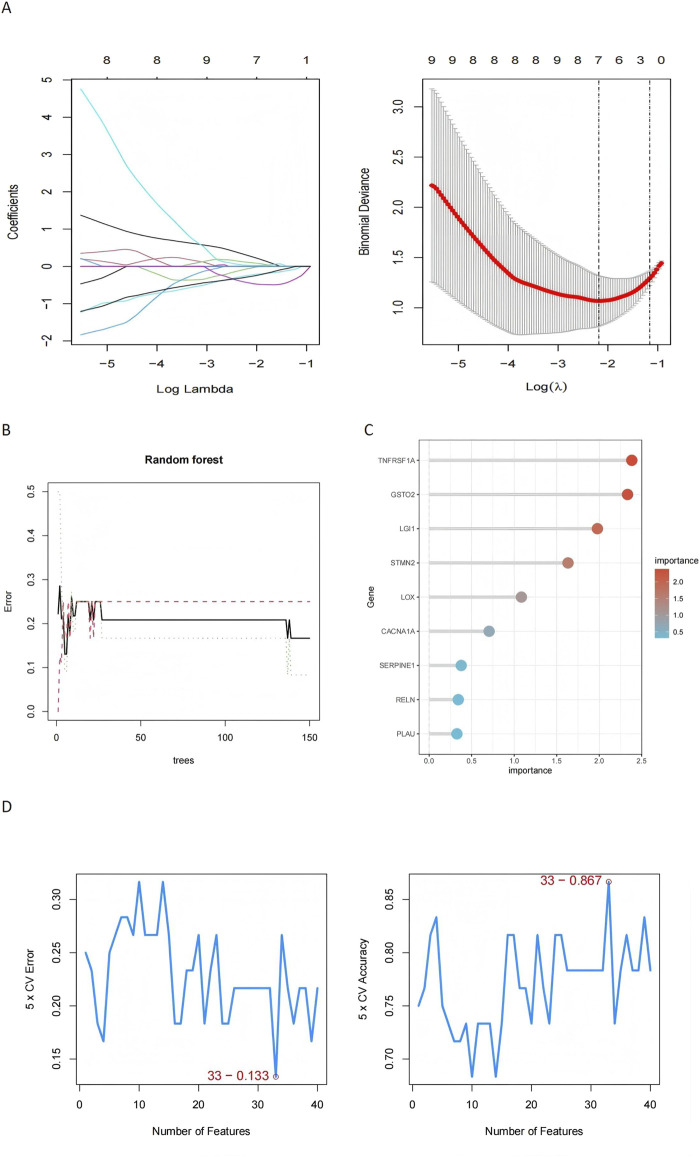
Machine learning-based identification of key genes in glioblastoma and degenerative CNS diseases. **(A)** The LASSO regression plot shows the coefficient profiles of the genes as the regularization parameter (log λ) changes, with the binomial deviance plot identifying the optimal λ value (log λ = −3), where 7 genes were selected based on cross-validation, demonstrating high predictive performance for distinguishing disease states. **(B)** Random Forest model performance is illustrated by the error plot, where the minimum error stabilizes after approximately 50 trees, and the importance scores of the top 9 genes. **(C)** The Random Forest gene importance plot further emphasizes the contributions of top genes. **(D)** The SVM-RFE results depict cross-validation error and accuracy as a function of the number of selected genes, with 33 features achieving the lowest error (0.133) and highest accuracy (0.867), demonstrating the effectiveness of this method in selecting a concise yet predictive subset of genes.

**FIGURE 7:**
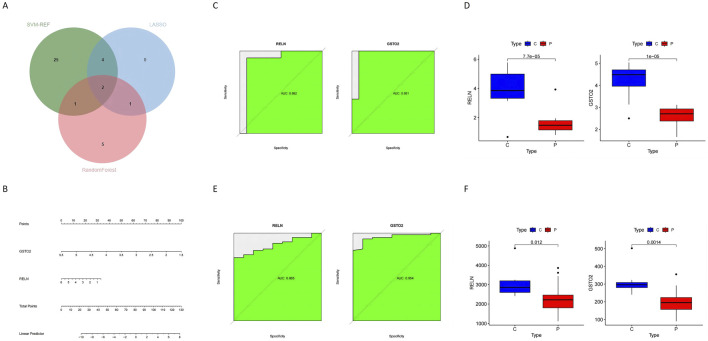
Identification and validation of hub genes in glioblastoma and degenerative CNS diseases **(A)** The Venn diagram shows the overlap of hub genes identified by three machine learning methods (SVM-RFE, Random Forest, and LASSO), revealing two common hub genes: *RELN* and *GSTO2*. **(B)** The nomogram prediction model integrates the two hub genes (*RELN* and *GSTO2*) to calculate the total points and predict the likelihood of disease, providing a practical clinical tool. **(C)** The ROC curves for *RELN* and *GSTO2* demonstrate their diagnostic performance in the training dataset, with AUC values of 0.982 and 0.981, respectively, indicating high accuracy. **(D)** Box plots show the expression levels of *RELN* and *GSTO2* in tumor (P) and control **(C)** groups, with both genes showing significantly higher expression in the control group (p < 0.0001). **(E)** ROC curves for *RELN* and *GSTO2* in the out-group validation dataset confirm their diagnostic performance, with AUC values of 0.895 and 0.894, respectively. **(F)** Box plots of the out-group validation dataset show consistent trends, with *RELN* and *GSTO2* expression levels significantly higher in the control group compared to the glioblastoma group (p = 0.012 and p = 0.014, respectively).

Machine learning approaches were used to refine the identification of key genes. LASSO regression selected seven genes using cross-validation (Figure 6A). Random Forest analysis identified nine genes based on feature importance (Figures 6B, C), while SVM-RFE highlighted 33 genes with an accuracy of 0.867 (Figure 6D). By intersecting the results of these methods, two genes, *RELN* and *GSTO2*, emerged as shared biomarkers between glioblastoma and degenerative CNS diseases (Figure 7A). These two genes showed robust potential for diagnostic applications and were further validated in subsequent analyses.

### 3.7 Validation of hub genes and regulatory network construction

**FIGURE 8:**
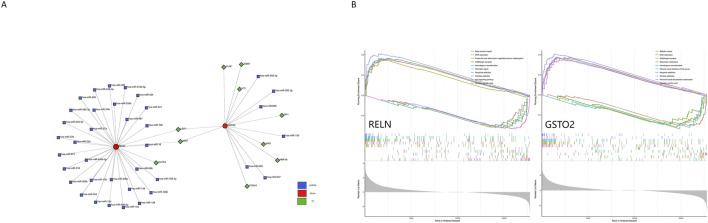
Regulatory network and pathway enrichment analysis of *RELN* and *GSTO2* in glioblastoma and degenerative CNS diseases. **(A)** The genes-TFs-miRNAs network illustrates the regulatory interactions among the two hub genes (*RELN* and *GSTO2*), their associated TFs, and miRNAs. **(B)** The ssGSEA analysis results for *RELN* and *GSTO2* reveal their involvement in key biological pathways.

To construct a hub gene nomogram model (Figure 7B), we assessed the diagnostic potential of *RELN* and *GSTO2* using ROC curve analysis. In the primary dataset, both genes achieved an AUC of 1, demonstrating excellent diagnostic accuracy (Figure 7C). The expression levels of *RELN* and *GSTO2* were significantly lower in glioblastoma samples compared to controls (Figure 7D). For external validation, we analyzed the GSE196533 dataset, where both genes also exhibited high diagnostic performance with AUC values exceeding 0.8 (Figure 7E). Similarly, expression levels of RELN and GSTO2 significantly decreased glioblastoma samples compared to controls (Figure 7F). Furthermore, regulatory network analysis, leveraging the ChEA and TarBase databases, identified key transcription factors and miRNAs that potentially interact with *RELN* and *GSTO2* (Figure 8A).

### 3.8 ssGSEA analysis

We performed single-gene GSEA analysis for each pivotal gene (*RELN* and *GSTO2*) and visualized the top five upregulated and downregulated pathways (Figure 8B). The results revealed that these genes are enriched in pathways such as DNA replication, GABAergic synapse, homologous recombination, and the synaptic vesicle cycle. Furthermore, they are associated with pathways related to various addictions, including morphine and nicotine addiction. These findings highlight the involvement of *RELN* and *GSTO2* in both CNS functions and broader physiological processes. However, it is important to note that GO analysis identifies commonly associated mechanisms and does not confirm specific functional relationships in the context of disease. Experimental validation is required to establish such relationships.

**FIGURE 9:**
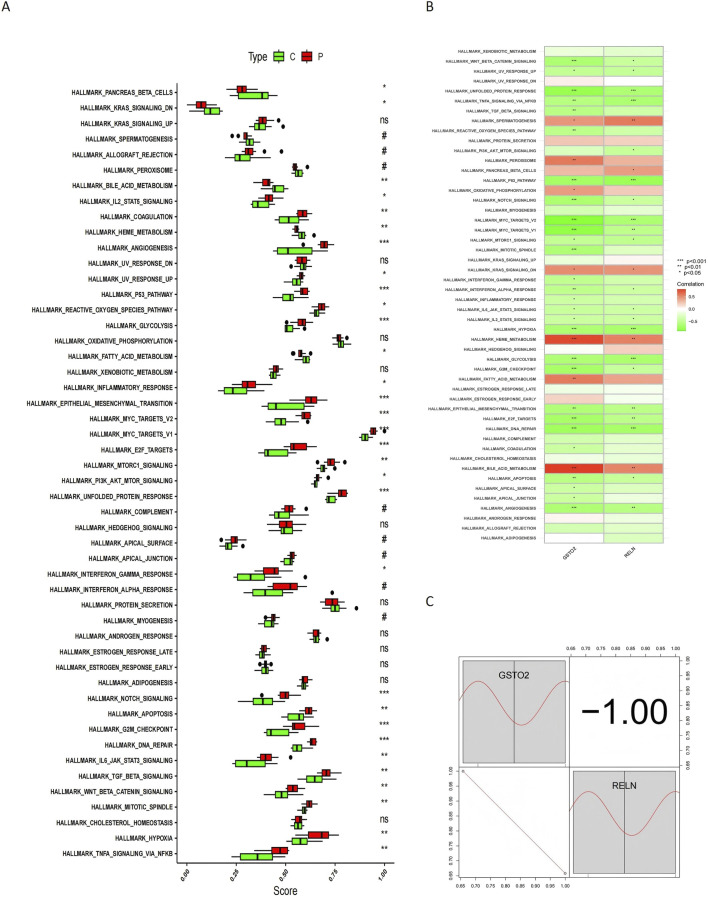
ssGSEA analysis and correlation of *RELN* and *GSTO2* with hallmark pathways. **(A)** Boxplots illustrate the differences in ssGSEA pathway enrichment scores between glioblastoma (P) and control **(C)** samples. **(B)** Heatmap displays the Spearman correlation analysis between hallmark pathways and hub gene expression levels. **(C)** Heatmap of gene-pathway correlations provides an intuitive depiction of the relationships between *RELN*, *GSTO2*, and hallmark pathways.

To further understand the expression patterns of these genes, boxplots were generated to illustrate their distribution across different sample groups (Figure 9A). Compared to control samples, glioblastoma samples exhibited increased expression of pathways such as coagulation, *p53* signaling, epithelial-mesenchymal transition, and *MYC* targets *V1/V2*. These pathways, which are linked to tumor progression, underscore the potential roles of *RELN* and *GSTO2* in glioblastoma biology.

We also examined the correlations between hub gene expression levels and hallmark pathways to explore potential regulatory linkages. Spearman correlation analysis revealed that *RELN* and *GSTO2* expression positively correlates with pathways such as spermatogenesis, heme metabolism, and oxidative phosphorylation, while negatively correlating with pathways like unfolded protein response, *TNFA* signaling *via NFKB*, *p53* signaling, and *MYC* targets (Figure 9B). These correlations provide insights into the functional roles of these genes in distinct biological processes and their potential involvement in cancer development. The relationship between these pathways and hub gene expression is further depicted in a heatmap for intuitive visualization (Figure 9C), supporting the hypothesis that *RELN* and *GSTO2* are critical mediators at the intersection of glioblastoma and degenerative CNS disease mechanisms.

## 4 Discussion

Nearly one-sixth of the world's population is affected by CNS diseases (Zhou et al., 2021). These disorders range from mild neurological impairment (which may manifest as motor, sensory, visual, speech, cognitive impairment, or a combination of these symptoms) to more severe conditions, such as coma and brain death. The central nervous system, including the brain and spinal cord, carries important tasks for processing sensory information, controlling movement, and coordinating all higher functions of the body, such as thinking, memory, and affect (Capani et al., 2016).

In many CNS diseases, ongoing progressive neurological damage is often caused by primary neuronal dysfunction. However, not all central nervous system disorders are degenerative. For example, although brain trauma or stroke affects the CNS, they are usually acute events and differ from chronic degenerative processes (Campbell and Khatri, 2020). In contrast, some CNS diseases such as Alzheimer's disease, Parkinson’s disease, and multiple sclerosis are indeed degenerative, and their main features are gradual deterioration in the structure and function of nerve cells, particularly neurons (Fan and Huo, 2021). These degenerative diseases progress over time, and damage and death of nerve cells lead to various dysfunctions. For example, cognitively function-associated neurons are particularly affected in Alzheimer's disease (Tiwari et al., 2019). Pathological mechanisms of these diseases may include factors such as abnormal protein folding, mitochondrial and energy metabolism disorders (Ferrer, 2018), oxidative stress (Singh et al., 2019), inflammation (Voet et al., 2019), and imbalances in neurotransmitter systems (Mahalakshmi et al., 2020). These complex pathological processes not only lead to the decline and/or death of neuronal function, but also further affect the overall function of the brain and spinal cord.

In 2014, GBM represented 16% of all primary brain and central nervous system tumors. The age-adjusted mean incidence rate was 3.19 per 100,000 (Thakkar et al., 2014). By 2023, however, the incidence of malignant brain tumors reached an alarming 7 cases per 100,000 and increased with age. The 5-year survival rate is approximately 36%. Older patients have a lower survival rate than younger patients. Survival rates ranged from approximately 71.5% in patients aged 15–39 years and 21% in patients aged 40 years and older. About 49% of these primary malignant brain tumors are glioblastomas (Schaff and Mellinghoff, 2023). Glioblastoma is a very aggressive brain tumor classified as grade IV in malignant glioma, which is the most severe grade. This tumor is characterized by rapid growth and high invasiveness to surrounding brain tissue. Symptoms of GBM include headache, seizures, neurocognitive impairment, and focal neurological deficits (Ostrom et al., 2022).

Recent research has unveiled a novel therapeutic strategy against cancer, drawing inspiration from treatments for neurodegenerative disorders (Soragni et al., 2016). This approach involves the development of ReACp53, a designed inhibitor that targets the aggregation of the *p53* protein, a common pathological feature in various cancers, notably ovarian carcinomas. By preventing *p53* aggregation, ReACp53 restores its tumor-suppressive function, leading to a reduction in tumor growth and an increase in cancer cell apoptosis. This breakthrough highlights the potential of leveraging neurodegenerative disease therapies to combat cancer, offering a promising avenue for future cancer treatment research.

Our study employed a novel approach that integrates WGCNA, multiple machine learning algorithms, and functional enrichment analyses to identify biomarkers shared between GBM and degenerative CNS diseases. This integrated pipeline offers several advantages over conventional methods.

First, traditional biomarker discovery methods often rely on a single dataset or apply univariate statistical methods to identify significant genes. These approaches may overlook subtle but biologically important interactions and fail to generalize across datasets. By contrast, our method combines data from multiple independent datasets, ensuring robustness and minimizing potential biases. The WGCNA framework, in particular, enables the identification of gene modules based on co-expression patterns, providing a systems-level understanding of disease-associated networks that traditional differential expression analyses cannot capture (Oluwatosin A et al., 2025).

To further refine the identification of key candidate genes from the WGCNA results, we integrated three machine learning techniques: SVM-RFE, LASSO, and Random Forest. Each method contributes unique strengths to the selection process. SVM-RFE is well-known for its ability to select relevant features while minimizing the risk of overfitting, making it particularly useful for analyzing datasets with a high number of variables (Hector et al., 2018). LASSO is effective in both feature selection and regularization, promoting models that are sparse, interpretable, and generalizable (Maike and Andreas, 2024). Random Forest, on the other hand, is highly precise in handling large datasets, managing data imbalance effectively, and providing valuable insights into feature importance (Ibrahim et al., 2024). By combining these methodologies, we capitalize on the unique strengths of each: LASSO’s simplicity and interpretability, SVM-RFE’s adaptability to complex datasets, and Random Forest’s comprehensive evaluation of feature relevance. This integrated strategy ensures thorough and reliable identification of crucial genes, enhancing the predictive capabilities and robustness of our analysis.

Traditional methods often neglect downstream functional validation at the pathway level, limiting the understanding of the broader biological context of identified biomarkers. In this study, we addressed this limitation by incorporating functional enrichment analyses (GO and KEGG) to link the identified biomarkers with specific biological processes and pathways. This approach not only strengthens the biological relevance of the biomarkers but also provides insights into their potential roles in disease mechanisms, paving the way for future experimental validation (Nguyen et al., 2024).

Building on these analyses, we further explored the biological context of the identified hub genes using the NetworkAnalyst platform, an online tool for comprehensive data analysis (Guangyan et al., 2019). This platform provided access to resources such as the ChEA database to predict TFs associated with the hub genes and the TarBase database to identify miRNAs linked to these genes. By integrating these analyses, our approach enhances the understanding of the pathways and regulatory networks in which the hub genes operate, shedding light on the potential mechanisms through which they influence disease processes.

When we delve into diseases of the central nervous system, we find unexpected associations between GBM and degenerative CNS diseases. Although there are clear differences in clinical presentation and pathogenesis between these two types of diseases, they show striking similarities in certain critical biological processes. Ferroptosis, as a key mode of cell death, plays a crucial role in both diseases. In degenerative neurological diseases such as PD, iron deposition and consequent oxidative damage are among the critical pathological processes (Martin-Bastida et al., 2017; Mitre et al., 2022). Further studies have shown that intervening in the metabolic pathway of iron can not only bring new therapeutic strategies for Parkinson’s disease (Wang et al., 2023), but also provide new ideas for the treatment of glioblastoma.

Lipid rafts serve as dynamic hubs for integrating signaling events in both tumor and neuronal cells, including those associated with *RELN* and Wnt signaling (Yunden and Ven, 2024). These lipid-rich microdomains play a pivotal role in organizing and coordinating the interaction of various signaling pathways, including the Wnt pathway, which intersects with *RELN* signaling through LRP6. This intersection is particularly significant in both tumor progression and neurodevelopmental processes (Lenz et al., 2024). In the case of tumors, including GBM, lipid rafts enable the integration of multiple pro-survival and growth-promoting signals, enhancing cellular responses to external stimuli (Reza et al., 2023; Pampa et al., 2024). Similarly, in neuronal cells, lipid rafts facilitate the *RELN*-mediated signaling crucial for processes like neuronal migration and synaptic plasticity, by coordinating the activation of downstream molecules such as *Dab1* (Matthew et al., 2022). Additionally, oxidative stress is modulated within these lipid domains, influencing cellular fate decisions by affecting both Wnt and *RELN* pathways (Akira et al., 2024). Alterations in lipid raft composition or function have been shown to disrupt this integration, contributing to both tumorigenesis (Momoko et al., 2023) and neurodegeneration (Yi Sak et al., 2024). Therefore, lipid rafts are indispensable for coordinating complex signaling networks in these vital biological processes.

While glioblastoma and degenerative central nervous system diseases differ fundamentally in their molecular mechanisms and pathological outcomes—such as cell death in degenerative CNS diseases (Salem et al., 2025) versus cell proliferation and resistance to cell death in cancer (Talvard-Balland et al., 2024)—our findings suggest that certain molecular pathways, such as those involving iron metabolism, may be shared (Brown et al., 2024). This observation broadens our understanding of these distinct neurological disorders and suggests potential overlapping therapeutic targets. Based on this, we investigated markers common to degenerative CNS diseases and GBM, identifying *RELN* and *GSTO2* as key candidates.

*RELN* is a large extracellular matrix protein first identified during brain development, crucial for regulating neuronal migration and cortical stratification. It involves processes like neuronal migration, dendritic outgrowth, dendritic spine formation, synapse generation, and synaptic plasticity (Khialeeva and Carpenter, 2017). Research has shown that *RELN* is expressed in the adult brain and plays roles in neuronal plasticity, memory formation, and various neurological diseases. Additionally, *RELN* impacts the immune system, liver fibrosis, and cancers (Canet-Pons et al., 2018).

In addition to their general role in cellular signaling, lipid rafts are essential for the precise regulation of the *RELN/ApoER2* signaling pathway, which is crucial for neuronal migration, synaptic plasticity, and neurodevelopment (Ling Xiao et al., 2024). These lipid-enriched microdomains serve as platforms that facilitate the clustering of signaling receptors such as *ApoER2*, enhancing the signal transduction efficiency. The interaction between *RELN* and *ApoER2* within lipid rafts has been shown to modulate the activation of downstream signaling molecules, including *Dab1*, a key kinase involved in neuronal migration (Mitsuki et al., 2024). Recent study had demonstrated that lipid rafts play a pivotal role in ensuring that this signaling cascade is both robust and specific, thereby facilitating the proper formation of neural networks (Harald et al., 2005). Moreover, lipid rafts may influence the trafficking and internalization of *ApoER2* receptors, which is necessary for effective signal initiation.

Disruption of lipid rafts can lead to a failure in receptor clustering and signaling efficiency, impairing essential neurodevelopmental processes like synaptogenesis and axon guidance. Emerging evidence suggests that such disruption could contribute to various degenerative CNS diseases by altering synaptic integrity and neuronal survival. Notably, research on AD and other neurodegenerative conditions has revealed that the perturbation of lipid raft domains can impair *RELN*/*ApoER2* signaling, resulting in deficits in memory, learning, and neuroplasticity (Christina M et al., 2024). Furthermore, alterations in lipid raft composition and function are thought to affect the balance between neuroprotection and neurodegeneration, implicating lipid rafts as a key player in both disease onset and progression. These findings underscore the critical role of lipid rafts not only in the *RELN*/*ApoER2* pathway but also in the overall maintenance of neuronal function and resilience (S Sakura et al., 2011).

*RELN* expression and function are crucial in degenerative CNS diseases such as AD, PD, and Huntington’s disease. Research indicates that *RELN* expression is typically decreased in these diseases, contributing to pathological processes such as the loss of neuronal connectivity, neuronal death, and cognitive decline. These findings suggest that *RELN*’s functional activity may be inhibited, further exacerbating disease progression. (Lidón et al., 2020). *RELN*’s mechanism involves neurodevelopment and neuronal plasticity. Core components of the *RELN* pathway include *VLDLR*, *ApoER2*, *Src* family kinases, and *Dab1* (Jossin, 2020). *RELN* binds to its receptors (*LRP1/2 and ApoER2/VLDLR*), activating downstream pathways like *Dab1*. This activation is essential for regulating intracellular calcium levels, reorganizing the cytoskeleton, and maintaining synapses (Bock and May 2016). In AD and other neurodegenerative disorders, reduced *RELN* signaling can alter synaptic structure and function, affecting neural network stability and plasticity, leading to cognitive decline. Reduced *RELN* signaling may impair NMDA receptor function, crucial for learning and memory (Joly-Amado et al., 2023).

The role of *RELN* in tumors is complex. Research shows that it plays a role in regulating the invasion and proliferation of cancer, and inhibits the migration and invasion of pancreatic cancer cells (Ramaker et al., 2017). The expression of *RELN* decreased in breast cancer, colorectal cancer and pancreatic cancer, but increased in retinoblastoma, myeloma and prostate cancer (Ndoye et al., 2021; Biamonte et al., 2021; Seigel et al., 2007; Qin et al., 2017). Some studies have found that the expression level of *RELN* in GBM is different from that of normal brain tissue or other types of brain tumors, which may be related to tumor invasiveness, growth rate, and treatment response. (Perrone et al., 2007). Moreover, recent studies have suggested that inhibiting heparanase, an enzyme involved in the remodeling of the extracellular matrix (Minghong et al., 2025), may significantly affect the regulation of autophagy and apoptosis in glioblastoma (Valeria et al., 2023). Heparanase inhibition has been shown to alter the balance between cell survival and cell death by modulating key signaling pathways involved in these processes (Vy M et al., 2017). By interfering with the autophagic flux and promoting apoptosis, heparanase inhibitors may enhance the sensitivity of glioblastoma cells to therapeutic agents, providing a promising approach for treatment strategies (Carolyn G and Renato V, 2022).

In GBM, the expression of *RELN* and its main downstream effector molecule *Dab1* is inhibited, and mRNA expression is inversely proportional to the degree of malignancy (Talebian et al., 2019). In addition, *RELN* expression was positively associated with survival in patients in two large independent clinical annotation datasets. Silencing of *RELN* occurs through promoter hypermethylation (Serrano-Morales et al., 2017). At the functional level, *RELN* regulates GBM cell migration in a *Dab1* (tyrosine phosphorylation) dependent and non dependent manner, depending on the substrate provided. Activation of *RELN* signaling significantly reduces the proliferation of GBM cells, a phenotype dependent on *RELN* stimulation of *Dab1*. Mutants lacking all *RELN* induced tyrosine phosphorylation sites (DAB1-5F) failed to induce growth arrest. Proteomic analysis shows that these effects are mediated by reducing the dephosphorylation of *E2F* targets and *ERK1/2*. *RELN* may also indirectly affect tumor growth and spread by affecting astrocytes and microglia in the tumor microenvironment. This interaction is achieved by altering the secretion of cytokines and chemokines, thereby affecting the interaction between tumor cells and their microenvironment (Jandial et al., 2017; Zeng et al., 2023).

*GSTO2* is a member of GST family, belonging to the Omega subfamily (Peng et al., 2023). This family plays a critical role in cellular detoxification processes. GST enzymes, found in animals, plants, and microorganisms, protect cells from oxidative stress and chemical damage by facilitating the conjugation of glutathione with various electrophilic compounds, including drugs, environmental toxins, and metabolic by-products, thereby promoting their excretion (Zhang et al., 2013).

Degenerative CNS diseases are often associated with increased oxidative stress and inflammation (Schmuck et al., 2005). Oxidative stress during these diseases can lead to cellular damage and death, accelerating disease progression. *GSTO2*, through its antioxidative activity, may help neutralize excess free radicals and reactive oxygen species, alleviating oxidative stress-induced damage to neurons and other brain cells. Studies suggest a significant correlation between *GSTO2* and the age of onset in AD and PD (Ozturk et al., 2005). Cha et al. (2023) found that knocking down the *GSTO2* gene in fruit flies leads to an age-related increase in *Cabeza* protein levels in neurons. *Cabeza*, a homolog of the human *FUS* protein associated with degenerative CNS diseases like ALS and Frontotemporal Dementia, shows increased mislocalization and aggregation in the cytoplasm of neurons and reduced solubility in aging neurons when *GSTO2* is knocked out. This suggests *GSTO2* plays a crucial role in regulating *Cabeza* localization and aggregation, potentially impacting neurodegenerative disease development (Machamer et al., 2018).

Abnormal expression of *GSTO2* is also related to the occurrence and development of tumors. Interestingly, *GSTO2* exhibits a bidirectional regulatory role in tumors. Several studies (Peng et al., 2023; Masoudi et al., 2009; Djukic et al., 2015) have discussed the association of *GSTO2* with cancers. Sumiya R (Sumiya et al., 2022) revealed that *GSTO2* is uniquely expressed in various lung stem cells but silenced in lung squamous cell carcinoma (LSCC) due to DNA hypermethylation. *GSTO2* regulates cell growth, *β-catenin* expression, and mitochondrial respiration through *p38* phosphorylation. Activation of *p38* MAPK by *GSTO2* leads to the downregulation of *β-catenin*, likely *via* ubiquitination, contributing to lung tissue homeostasis and preventing malignant transformation. Loss of *GSTO2* in LSCC disrupts this mechanism, allowing *β-catenin* to avoid degradation, leading to increased mitochondrial oxidative phosphorylation and enhanced energy production, promoting LSCC growth. These findings highlight the potential importance of *GSTO2* in preventing LSCC and provide insights into lung carcinogenesis pathways. However, the specific role and mechanism of *GSTO2* in GBM require further investigation. It is hypothesized that *GSTO2*, by regulating oxidative stress responses and metabolic processes, could influence GBM cell survival, proliferation, and treatment response. Upregulation of *GSTO2* might enhance tumor cells’ ability to clear chemotherapeutic drugs, reducing treatment efficacy. Conversely, reducing *GSTO2* activity or expression could increase oxidative stress, promoting cancer cell death, and potentially serving as a therapeutic strategy.

Although *RELN* and *GSTO2* have different functions and regulatory mechanisms, studies suggest they may have potential interrelated mechanisms in certain diseases. *RELN* plays a crucial role in neural development and synaptic plasticity, and its abnormal expression is associated with degenerative CNS diseases. *RELN* combats oxidative stress by regulating synapse formation and stability (Sajjad et al., 2014), while *GSTO2* is important in the cellular antioxidant response, helping to neutralize excess free radicals and ROS, thereby reducing neuronal damage (Lauren E et al., 2019). *RELN* and *GSTO2* may jointly regulate oxidative stress and inflammatory responses in neurons, protecting them from damage. Additionally, *RELN* affects the stability and plasticity of neural networks by regulating synapse structure and function (Murat S et al., 2009), whereas *GSTO2*, through its antioxidant activity, reduces apoptosis and autophagy induced by oxidative stress, thus protecting neuronal survival. They may co-operate in the pathways of neuronal apoptosis and autophagy, maintaining normal neuronal function and survival (Xifeng et al., 2022). The signaling pathways involving *RELN* and *GSTO2* may interact at certain points; for instance, *RELN* influences intracellular calcium levels by regulating synaptic function and stability (Yehui et al., 2019), while *GSTO2* maintains cellular homeostasis by mitigating oxidative stress (Tatjana et al., 2022). They may influence each other in cellular signal transduction, jointly maintaining neuronal function and survival.

Clinically, our findings indicate that *RELN* and *GSTO2* show significant expression changes in GBM, suggesting their potential utility as biomarkers for this disease. Specifically, the observed differential expression of these genes in GBM samples compared to controls highlights their diagnostic relevance. However, we acknowledge that further experimental and clinical validation is essential to substantiate their roles in early diagnosis, disease progression monitoring, and treatment response evaluation.

Regarding neurodegenerative CNS diseases, we did not evaluate *RELN* and *GSTO2* in relevant cohorts; thus, their potential roles in neurodegenerative CNS diseases remain speculative. While shared molecular mechanisms between neurodegenerative CNS diseases and GBM suggest possible connections, we refrain from making definitive claims about their biomarker or therapeutic potential in these diseases without supporting evidence. Future research should validate these findings in disease-specific cohorts and investigate whether *RELN* and *GSTO2* could serve as progression markers or therapeutic targets for GBM and other CNS diseases.

## 5 Conclusion

This study involves collecting cross-gene data for AD, PD, MS, and ALS from the GeneCards database, and performing differential expression analysis of GBM using GEO datasets. Machine learning is used to analyze these data, aiming to find common molecular biomarkers between degenerative CNS diseases and GBM. This approach could uncover novel biomarkers and shared pathological processes in these disorders, guiding future therapy strategies. The study integrates molecular characteristics with clinical features to improve understanding of these disorders and to identify molecular targets for diagnosis, monitoring, and treatment. The results could also support personalized medicine based on molecular mechanisms. In this study, we identified co-expressed biomarkers for degenerative CNS diseases and GBM: *RELN* and *GSTO2*. Validated experimental results indicated that, in GBM patient samples, the expression levels of *RELN* and *GSTO2* were significantly reduced compared to healthy controls. Based on these findings, we hypothesize that *RELN* and *GSTO2* may act as protective factors against the development of these neurological disorders.

Overall, the study seeks to advance the diagnosis, treatment, and prognosis of degenerative CNS diseases and GBM by identifying biomarkers, using cross-disease analysis, and applying machine learning, potentially benefiting patients.

## Data Availability

The original contributions presented in the study are included in the article/Supplementary Material, further inquiries can be directed to the corresponding author.

## Supplementary material

SUPPLEMENTARY TABLE S1
DEGs.

SUPPLEMENTARY TABLE S2
Genes of WGCNA.

SUPPLEMENTARY TABLE S3
Degenerative CNS diseases.
