# Supplementary material for: Biomarkers in glioblastoma and degenerative CNS diseases: defining new advances in clinical usefulness and therapeutic molecular target
Source: Front Mol Biosci. 2025 Mar 18;12:1506961. doi: 10.3389/fmolb.2025.1506961 (PMC11959069; doi:10.3389/fmolb.2025.1506961)
Supplement: Supplementary file 1 [file Table1.docx]

Abbreviations: AD = Alzheimer 's disease, PD = Parkinson' s disease, ALS = amyotrophic lateral sclerosis, MS = multiple sclerosis, GBM = glioblastoma, CNS = central nervous system, LSCC = lung squamous cell carcinoma, WGCNA = Weighted Gene Co-expression Network Analysis, LASSO = lasso regression algorithm, SVM-REF = support vector machine-recursive feature elimination, DEG = differentially expressed gene, KEGG = Kyoto Encyclopedia of Genes and Genomes, GO = Gene Ontology, ROC = receiver operating characteristic, TOM = topological overlap matrix, AUC = area under curve, ssGSEA = single sample gene set enrichment analysis, CC = cellular component, MF = molecular function, BP = biological process = ssGSEA = single sample gene set enrichment analysis.
